# Seaweed Polysaccharides (Laminarin and Fucoidan) as Functional Ingredients in Pork Meat: An Evaluation of Anti-Oxidative Potential, Thermal Stability and Bioaccessibility

**DOI:** 10.3390/md13042447

**Published:** 2015-04-20

**Authors:** Natasha C. Moroney, Michael N. O’Grady, Sinéad Lordan, Catherine Stanton, Joseph P. Kerry

**Affiliations:** 1 Food Packaging Group, School of Food and Nutritional Sciences, College of Science, Engineering and Food Science, University College, Cork, Ireland; E-Mails: natasha.moroney@gmail.com (N.C.M.); Michael.OGrady@ucc.ie (M.N.G.); 2 Teagasc Food Research Centre, Moorepark, Fermoy, Co. Cork, Ireland; E-Mails: s.lordan@ucc.ie (S.L.); Catherine.Stanton@teagasc.ie (C.S.)

**Keywords:** laminarin, fucoidan, seaweed extract, *in vitro* digestion, bioaccessibility, pork

## Abstract

The anti-oxidative potential of laminarin (L), fucoidan (F) and an L/F seaweed extract was measured using the DPPH free radical scavenging assay, in 25% pork (*longissimus thoracis et lumborum* (LTL)) homogenates (TBARS) (3 and 6 mg/mL) and in horse heart oxymyoglobin (OxyMb) (0.1 and 1 mg/mL). The DPPH activity of fresh and cooked minced LTL containing L (100 mg/g; L_100_), F_100_ and L/F_100,300_, and bioaccessibility post *in vitro* digestion (L/F_300_), was assessed. Theoretical cellular uptake of antioxidant compounds was measured in a transwell Caco-2 cell model. Laminarin displayed no activity and fucoidan reduced lipid oxidation but catalysed OxyMb oxidation. Fucoidan activity was lowered by cooking while the L/F extract displayed moderate thermal stability. A decrease in DPPH antioxidant activity of 44.15% and 36.63%, after 4 and 20 h respectively, indicated theoretical uptake of L/F antioxidant compounds. Results highlight the potential use of seaweed extracts as functional ingredients in pork.

## 1. Introduction

Seaweed polysaccharides (laminarin and fucoidan) isolated from the cell walls of brown seaweed (*Laminaria digitata*) possess immunomodulatory, anti-inflammatory, antiviral, antitumor, antithrombotic anticoagulant and antioxidant bioactivities [1,2]. Structurally, laminarin is composed of β-(1,3)-linked glucose containing large amounts of sugars and a low fraction of uronic acids. Two types of polymeric chains are present in laminarin, G-chains with glucose at the end and M-chains with mannitol as the terminal reducing end [3]. The antioxidant activity of laminarin has been linked to molecular structure, degree and length of branching and the monosaccharide constituents [4]. The structure of fucoidan consists mainly of α(1,3)-linked l-fucopyranose residues with sulphates at the C-2 position [5]. Distinct conclusions regarding chemical structures of fucoidans are often difficult to formulate due to structural heterogeneity and lack of regularity in fucoidan molecules [6]. Sulphate content, degree of sulphation and molecular weight are often attributed as factors influencing the antioxidant activity of fucoidan [7].

A wide range of analytical techniques (e.g., HPLC, ATR-FTIR and NMR spectroscopy) may be used to characterise and quantify structurally complex polysaccharides, such as laminarin and fucoidan, present in seaweeds [8]. Such techniques can involve detailed and time consuming extraction, preparation and sample clean-up procedures, depending on the parent seaweed material or the matrix in which the compounds of interest (polysaccharides) are contained [9]. *In vitro* antioxidant assays (e.g., FRAP, ABTS, ORAC and DPPH free radical scavenging activities) are frequently used to assess the antioxidant activity and potency of plant extracts [10]. The DPPH assay (based on a quick electron transfer reaction, followed by a slower hydrogen transfer reaction) is a simple, rapid, sensitive and reproducible index of antioxidant activity [11]. DPPH free radical scavenging activity of seaweed extracts, including laminarin and fucoidan, has been reported for a number of seaweed species [11,12].

The addition of antioxidant compounds to muscle foods (via the animals’ diet or direct addition) in order to enhance meat quality and shelf-life has attracted much research attention in recent years. Previous research indicated that functional ingredients, such as laminarin and fucoidan, have beneficial effects pre-(animal health) [13] and post-slaughter (meat quality) [14]. Moroney *et al.* [15] reported that the addition of seaweed extracts, containing laminarin and fucoidan, to pig diets, resulted in lower levels of lipid oxidation in fresh pork steaks. However, direct addition of the same seaweed extract, promoted lipid oxidation and decreased the surface redness of fresh pork patties [16]. Catalysis of lipid oxidation was linked to the presence of salt and minerals in the seaweed extract. Increased discolouration (oxymyoglobin oxidation) was attributed to the effect of oxidising lipids and potential interactions between seaweed polysaccharides and oxymyoglobin. The anti- and pro-oxidative activity of laminarin and fucoidan on lipid and oxymyoglobin oxidation processes will be further examined in the present study.

The chemical structure of plant cell wall polysaccharides (e.g., cellulose, pectin substances, inulin and gums) and other associated non-carbohydrate components (*i.e.*, resistant protein) can be sensitive to chemical, mechanical, thermal and enzymatic processing [17]. Therefore the consequence of cooking on the potential bioactivity of laminarin and fucoidan in a meat matrix should be considered when formulating a functional meat product [18]. Cooking may sometimes improve the antioxidant activity of plant based materials due to the formation of other antioxidant components such as Maillard reaction products (MRPs) [19]. MRPs have been reported to possess antiradical activity including inhibition of the DPPH, oxygen peroxyl and hydroxyl radicals as well as copper and Fe^2+^ chelators [20]. In a previous study, Moroney *et al.* [16] reported a reduction in lipid oxidation of cooked minced pork patties containing laminarin and fucoidan which was attributed partially to the cooking process and the formation of MRPs which were not present in the fresh pork patties.

The digestion process may influence the bioactivity and bioaccessibility of laminarin and fucoidan. Bioaccessibility is defined as the fraction of a compound transferred from the food matrix during digestion, and thus made accessible for intestinal absorption and cellular uptake [21]. *In vitro* digestion models provide a useful alternative to animal and human models and simulate the digestion process of the human gastrointestinal tract (GIT). Cell culture models, in particular the Caco-2 cell culture model, have been widely utilised as part of *in vitro* digestion models as a predictive tool for the absorption of bioactive compounds from foods [22].

Studies on the anti-oxidative potential of seaweed polysaccharides in meat products are limited and merit investigation. Furthermore, the literature lacks information regarding the bioaccessibility of seaweed polysaccharides in meat products after cooking and post digestion. The initial objective of this study was to profile the antioxidant activity of laminarin (L), fucoidan (F) and a seaweed extract containing L and F, using the DPPH free radical scavenging assay. The antioxidative potential of L, F and L/F was further examined in fresh pork *longissimus thoracis et lumborum* (LTL) homogenates and in commercial horse heart oxymyoglobin. The DPPH radical scavenging and thermal stability of L, F and L/F in cooked pork patties was assessed. Finally cooked pork patties were subjected to an *in vitro* digestion procedure to determine the effects of digestion on the antioxidant potential of L, F and L/F and L/F digestates were examined in a transwell Caco-2 cell model to assess theoretical cellular uptake of antioxidant components of L/F.

## 2. Results and Discussion

### 2.1. Free Radical Scavenging Activity of Seaweed Polysaccharides (L, F and L/F)

In general, the DPPH free radical scavenging activity of seaweed polysaccharides increased over 20 h and followed the order: Trolox > F_1_ > L/F_3_ > L/F_1_ > L_10_ ≈ L_1_ (Table 1). DPPH free radical scavenging activity of L/F increased as a function of concentration. The DPPH free radical scavenging activities reported for L_1_ and L_10_ were comparable to previously reported values (1.4%–5.3%) for laminarin extracted from *Laminaria digitata* at concentrations ranging from 0.125 to 1.0 mg/mL [12]. The DPPH free radical scavenging activity of F_1_ (66.13%) after 1 h in the present study was similar to the inhibition of the DPPH radical (55.22%) after 30 min by fucoidan (1 mg/mL) from Sigma reported by Mak *et al.* [7].

Limited research suggests that carbohydrate polymers such as β-glucans (laminarin) possess free radical scavenging activity, however the addition of high levels of β-glucans is often necessary before radical scavenging activity is observed [23,24]. At concentrations of 20–200 mg/mL (higher than those used in the present study) a 1,3 β-d-glucan enriched extract from cereal grains demonstrated 25%–80% inhibition of the DPPH radical [23]. The mechanism of antioxidant action of β-d-glucans against free radicals is still not well understood, but a number of theories exist [25]. Tsiapali *et al.* [24] reported enhanced antioxidant activity of laminarin polymers over monomeric units due to greater ease of abstraction of anomeric hydrogen from one of the internal monosaccharide units rather than from the reducing end. In the present study, laminarin exhibited weak radical scavenging activity which may be due to the level examined.

**Table 1:** Free radical scavenging activity (DPPH) of L, F and L/F for up to 20 h at ~20 °C.

Incubate	Time, h		
	1	4	20
L_1_ *	1.09 ± 0.92 ^ab^	1.39 ± 0.96 ^ab^	1.64 ± 1.30 ^ab^
L_10_	1.55 ± 1.21 ^b^	2.72 ± 1.77 ^b^	3.16 ± 2.72 ^b^
F_1_	66.13 ± 0.32 ^c^	76.48 ± 0.30 ^c^	90.68 ± 0.55 ^c^
L/F_1_	35.43 ± 2.04 ^d^	47.35 ± 1.79 ^d^	69.51 ± 1.37 ^d^
L/F_3_	56.18 ± 1.01 ^e^	68.40 ± 0.89 ^e^	78.41 ± 0.21 ^e^
Trolox	95.89 ± 0.08 ^f^	95.92 ± 0.14 ^f^	95.76 ± 0.48 ^f^

* Subscripts 1, 3 and 10 denote concentrations in mg/mL; ^a–f^ Within each storage time, mean values (± standard deviation) in the same column bearing different superscripts are significantly different, *p* < 0.05.

For some antioxidants, such as Trolox, the reaction with DPPH is rapid while other compounds may react more slowly [26]. The ability of seaweed extracts to quench free radicals is known to take place over longer periods of time compared to rapid acting synthetic antioxidants such as butylated hydroxyanisole (BHA) [27,28]. Slower reacting compounds are hypothesised to have a more complex reaction mechanism involving one or more secondary reactions in quenching the DPPH radical [10]. In the present study, after 20 h, the DPPH free radical scavenging activity of F_1_ was equivalent (although statistically lower) to the positive control (Trolox), and significantly (*p* < 0.05) higher than both L_1_ and L_10_. Therefore the ability of an antioxidant to reduce and quench free radicals over a longer period of time may have benefits for extending the shelf-life of processed foods [28].

### 2.2. Effect of Seaweed Polysaccharides on Lipid Oxidation in Pork Muscle Model Systems

**Figure 1:**
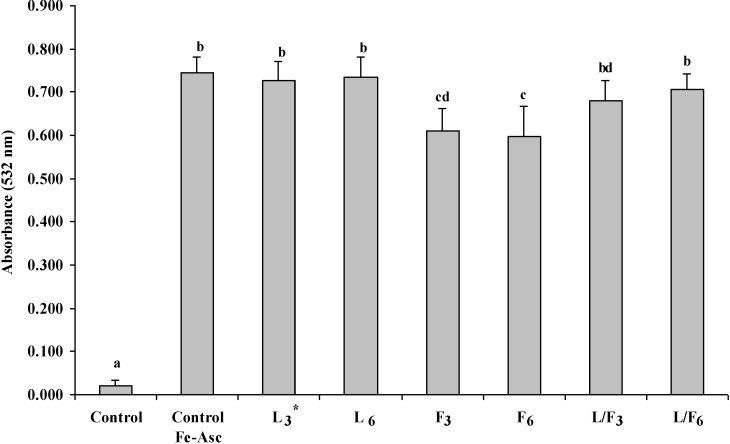
Lipid oxidation in 25% *longissimus thoracis et lumborum* (LTL) pork muscle homogenates following the addition of L, F or L/F and storage for up to 4 h at 4 °C. * Subscripts 3 and 6 denote concentrations in mg/mL. ^abcd^ Mean values (± standard deviation error bars) bearing different superscripts are significantly different, *p* < 0.05.

*In vitro* antioxidant assays (e.g., the DPPH assay) highlight the potential antioxidant activities of compounds but may not accurately predict activity in complex test systems such as muscle foods. To further investigate antioxidant activities of L, F and L/F, pork meat homogenates were subjected to iron/ascorbate (FeCl_3_/sodium ascorbate)-induced lipid oxidation. Compared to the control, after 4 h at 4 °C, lipid oxidation significantly increased (*p* < 0.05) in the pork meat homogenates with the addition of pro-oxidants (Figure 1). No difference was observed for L_3_ and L_6_ compared to the control. Similarly no inhibition of lipid oxidation by laminarin, at levels comparable to those in the present study (3 mg/mL), was observed in a linoleic acid emulsion system [25]. F_3_ and F_6_ significantly decreased (*p* < 0.05) levels of lipid oxidation in pork meat homogenates. Trends indicated that levels of lipid oxidation in L/F_3_ and L/F_6_ were lower than the control (with pro-oxidants) although results were not statistically significant. In a previous study, Moroney *et al.* [16] reported that salt and minerals, present in the L/F extract, may have promoted lipid oxidation in fresh pork patties. Minerals and salt present in L/F_3_ and L/F_6_ may have counteracted the antioxidant activity of other constituents in the extract, thus impeding ability to significantly enhance lipid stability in the pork meat homogentates (Figure 1).

Structurally laminarin does not contain sulphate groups, which reportedly increases the antioxidant activity of fucoidan [29]. Sulphate groups can enhance the steric hindrance between polymer chains in polysaccharides leading to a more ordered and expanded conformation thus improving homogeneity in aqueous solution [30]. Lower molecular weight polysaccharides are often linked to increased free radical scavenging ability, presumably due to a non-compact structure which may allow more available sulphate and hydroxyl groups react with free radicals [9]. However, this was not observed for L in the present study indicating that even at low molecular weight, the structure in the presence of pork meat was unable to inhibit lipid oxidation, similar to the lack of DPPH free radical scavenging activity observed in Section 2.1 (Table 1).

In general, it is accepted that natural antioxidants scavenge free oxygen-centered radicals via two major mechanisms, hydrogen atom transfer (HAT) reactions and electron transfer (ET) reactions. Yan *et al.* [30] suggested the HAT reaction is more likely to occur in neutral polysaccharides, such as laminarin, while the ET is the probable mechanism in acidic polysaccharides, like fucoidan where the negative charge of the sulphate groups plays a large part in the radical scavenging activity. In the present study, fucoidan is most likely responsible for the antioxidant activity observed by the L/F extract in the pork meat homogenates presumably due to ET reactions between the sulphate groups and the free radicals in the pork meat homogenates.

### 2.3. Effect of Seaweed Polysaccharides on Oxymyoglobin Oxidation

**Figure 2:**
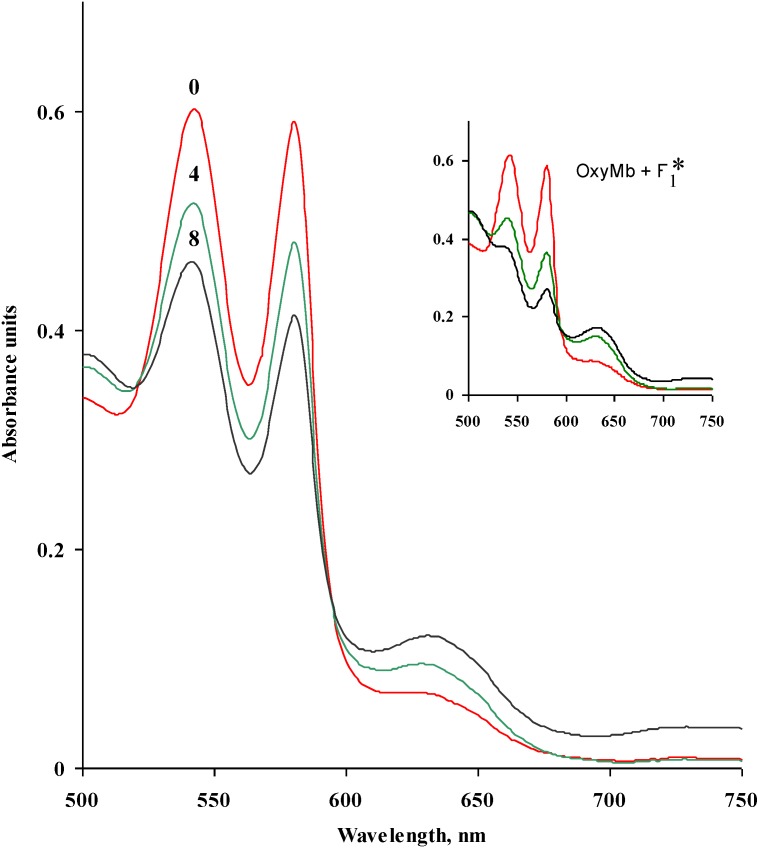
Absorbance spectra of oxymyoglobin (OxyMb) alone and following the addition of F_1_ (* Subscript 1 denotes concentration in mg/mL) and storage for up to 8 days at 4 °C.

Oxymyoglobin oxidation (represented by a reduction in OxyMb, %) increased during storage for up to 8 days at 4 °C (Table 2). L_0.1_ and L_1_ had no influence on OxyMb oxidation, however F_0.1_ and F_1_ significantly (*p* < 0.05) enhanced OxyMb oxidation compared to the control in a dose dependant manner on days 4 and 8 of storage. Similarly, a significant increase (*p* < 0.05) in OxyMb oxidation was observed for L/F_0.1_ and L/F_1_. The presence of metmyoglobin is characterised by an increased absorption at ~628 nm [31] which is evident in the spectral scan for OxyMb alone and OxyMb + F_1_ (Figure 2). At the wavelengths examined, no spectral shift in the presence of F_1_ was observed.

**Table 2:** Oxymyoglobin (OxyMb) oxidation (represented by a reduction in OxyMb) following the addition of L, F or L/F and storage for up to 8 d at 4 °C.

Incubate	time, d		
	0	4	8
Control	76.53 ± 2.28 ^a^	59.92 ± 2.30 ^a^	54.46 ± 2.02 ^a^
L_0.1_ *	76.57 ± 2.31 ^a^	59.68 ± 2.14 ^ab^	54.00 ± 2.50 ^a^
L_1_	76.59 ± 2.73 ^a^	58.11 ± 3.12 ^abc^	52.51 ± 2.75 ^a^
F_0.1_	74.73 ± 2.54 ^ac^	53.44 ± 2.44 ^bd^	45.42 ± 2.56 ^b^
F_1_	67.55 ± 2.50 ^b^	32.95 ± 2.00 ^e^	21.71 ± 1.34 ^c^
L/F_0.1_	74.93 ± 2.06 ^ad^	52.91 ± 2.44 ^cd^	44.95 ± 2.82 ^b^
L/F_1_	69.03 ± 2.78 ^bcd^	39.01 ± 1.90 ^e^	28.78 ± 2.25 ^d^

* Subscripts 0.1 and 1 denote concentrations in mg/mL; ^abcde^ Within each storage time, mean values (± standard deviation) in the same column bearing different superscripts are significantly different *p* < 0.05.

The exact mechanism by which fucoidan promotes OxyMb oxidation is unclear. The ability of fucoidan to bind to proteins such as antithrombin (a glycoprotein) and bovine serum albumin (a globular protein) has previously been linked to molecular weight as well as the sulphation patterns of the polysaccharide [32,33,34]. Generally, interactions between anionic polysaccharides and positively charged OxyMb have been reported to be electrostatic in nature due to opposing charges [35].

Similarly, Satoh *et al.* [36] demonstrated that oxidation of OxyMb was initiated via nucleophilic attack at the iron (II) centre of OxyMb by a water molecule with strong proton assistance from the distal histidine, or a hydroxide anion (OH^−^). These reactions can cause irreversible displacement of bound dioxygen from OxyMb resulting in the formation of ferric metmyoglobin and generation of the superoxide anion. In the present study, the anionic sulphate groups of fucoidan potentially enhanced the oxidation of OxyMb through the nucleophilic displacement mechanism described above.

### 2.4. Effect of Cooking on the DPPH Free Radical Scavenging Activity of Seaweed Polysaccharides in Pork Meat

**Figure 3:**
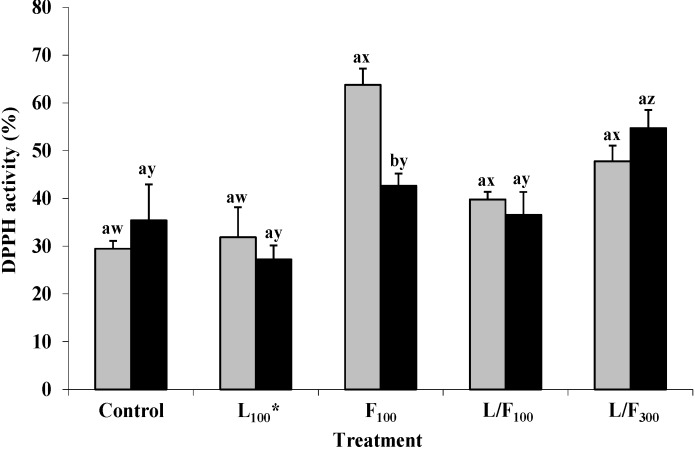
Free radical scavenging activity (DPPH) of L, F or L/F in fresh and cooked minced *longissimus thoracis et lumborum* (LTL) pork muscle stored for 20 h at ~20 °C. * Subscripts 100 and 300 denote concentrations in mg/g. ^ab^ Within each treatment, mean values (± standard deviation error bars) bearing different superscripts are significantly different, *p* < 0.05. Comparing ^wx^ fresh and ^yz^ cooked LTL pork muscle treatments to their respective controls, mean values bearing different superscripts are significantly different, *p* < 0.05. (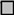), fresh; (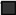), cooked.

Statistical analysis indicated that the DPPH free radical scavenging of L, F and L/F in the presence of fresh minced LTL (F_100_ > L/F_300_ ≈ L/F_100_ ≈ L_100_) followed a similar pattern to the DPPH free radical scavenging activities of seaweed polysaccharides reported in Section 2.1. L_100_ DPPH free radical scavenging was similar to the control before and after cooking (Figure 3). The DPPH free radical scavenging activity of F_100_ significantly (*p* < 0.05) decreased after cooking. Thermal processing is known to modify the physicochemical properties of plant cell wall polysaccharides [17]. The DPPH free radical scavenging activities of fresh and cooked L/F_100_ and L/F_300_ were similar indicating moderate thermal stability of the L/F extract. Similarly, Moroney *et al.* [14] reported low to moderate thermal stability of L/F in cooked minced pork patties from pigs fed the L/F extract for 3 weeks pre-slaughter.

L/F_300_ significantly (*p* < 0.05) enhanced the DPPH free radical scavenging activity of cooked minced LTL compared to the control (Figure 3). Similarly, Prabhasankar *et al.* [37] reported an increase in DPPH free radical scavenging activity of cooked pasta with the addition of a brown seaweed (*Undaria pinnatifida*) to uncooked pasta. The formation of Maillard reaction products (MRP) and other novel antioxidant compounds such as mycosporine-like amino acids during heat treatment of seaweed extracts has been reported [38,39,40]. Additionally, MRP have proven effective inhibitors of lipid oxidation in cooked minced pork patties [41]. In the present study, MRP formed during heating of L/F_300_ most likely enhanced the DPPH free radical scavenging of cooked minced LTL.

### 2.5. DPPH Free Radical Scavenging Activity of Seaweed Polysaccharides in Pork Meat Following in Vitro Digestion

**Figure 4:**
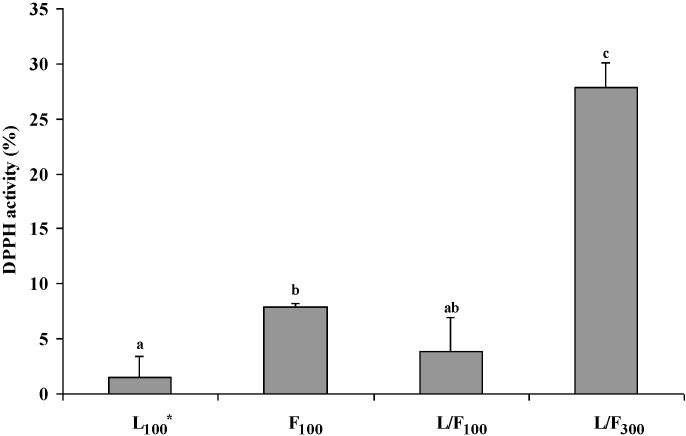
Free radical scavenging activity (DPPH) of L, F or L/F in digested cooked minced *longissimus thoracis et lumborum* (LTL) pork muscle stored for 20 h at ~20 °C. * Subscripts 100 and 300 denote concentrations in mg/g. ^abc^ Mean values (± standard deviation error bars) bearing different superscripts are significantly different, *p* < 0.05.

During the digestion procedure, cooked minced LTL from each treatment was subjected to pH changes and enzymatic reactions which resulted in increased (~30%–44%) DPPH free radical scavenging activities in digestates compared to undigested aqueous fractions (data not shown). The DPPH free radical scavenging activity of the control post digestion increased from 14.4% to 44.8% and was attributed to the presence of compounds such as peptides released from the pork meat during the *in vitro* digestion procedure. Escudero *et al.* [42] reported 51 different peptides were released from pork meat (*longissimus dorsi*) following *in vitro* digestion. Additionally, peptides obtained from animal sources such as porcine myofibrillar proteins have demonstrated DPPH free radical scavenging activity [43,44,45]. Data from each treatment (L_100_, F_100_, L/F_100_ and L/F_300_) were adjusted for the meat control to estimate the antioxidant activity due to the seaweed polysaccharides post digestion (Figure 4).

The DPPH free radical scavenging activity of digested L_100_ and L/F_100_ were similar (Figure 4). Laminarin is resistant to digestion in the upper GIT including acidic and enzymatic hydrolysis [46]. Salyers *et al.* [47] established two different types of enzymes (laminarases and β-glucosidases) were essential to fully degrade laminarin and were only synthesised after 4–6 h of incubation in the presence of the inducer. In the present study, the lack of suitable enzymes to break down laminarin in the *in vitro* digestion model used may explain the lack of enhanced antioxidant activity post digestion.

F_100_ and L/F_300_ significantly (*p* < 0.05) enhanced the DPPH free radical scavenging activity of cooked minced LTL post digestion. A few fucan-degrading enzymes have been obtained from marine bacteria and molluscs, however complete enzymatic breakdown has not been reported. The presence of sulphate groups attached to fucoidan has been postulated as a reason for resistance to enzymatic breakdown during digestion. The retention of the sulphate groups during digestion results in high ionic exchange capacities such as the binding of bile salts and scavenging of free radicals throughout the GIT before potential absorption [48]. The enhanced DPPH radical scavenging activity of F_100_ and L/F_300_ in cooked minced LTL, in the present study, may be due to the retention of the sulphate groups throughout the *in vitro* digestion procedure.

The DPPH free radical scavenging activity of digested L/F_300_ was significantly (*p* < 0.05) greater than F_100_. Fucoidan may be partially responsible for the scavenging activity of the extract. The synergistic effect between components in the L/F extract, such as protein and mannitol, could have contributed to the observed enhanced free radical scavenging activity in cooked minced LTL post digestion. Antioxidant activity, post-digestion, of bioactive peptides extracted from seaweeds has been reported previously [49]. Mannitol is frequently considered as a reference for carbohydrate-type antioxidants due to its established scavenging abilities [24]. Additionally, MRPs formed during cooking may have enhanced the DPPH free radical scavenging activity of L/F_300_ post digestion.

### 2.6. Bioaccessibility of Seaweed Polysaccharides in Pork Meat after Incubation with Caco-2 Cells

The aqueous fraction of the control and L/F_300_ digestates was incubated with Caco-2 cells for 4 and 20 h to determine the bioaccessibility of L/F post digestion. The DPPH free radical scavenging activity of L/F_300_, post digestion, was 56.49% higher than the meat control. Following incubation of the control and L/F_300_ digestates with Caco-2 cells for 4 and 20 h, the DPPH free radical scavenging activity of L/F_300_ was 12.34% and 19.85% higher than the meat control, respectively. The reduction in the DPPH free radical scavenging activity indicated theoretical uptake of some compounds with antioxidant activity. Therefore theoretical cellular uptake of seaweed polysaccharides was 44.15% and 36.63% (DPPH free radical scavenging activity) after incubation with Caco-2 cells at 4 and 20 h, respectively. Similarly, Soler-Rivas *et al.* [50] reported a decrease in ABTS free radical activity after digested grilled mushrooms were incubated with Caco-2 cells. Previously reported studies indicated that seaweed polysaccharides can be, to some extent, absorbed into the blood stream post digestion; however metabolism of these components after absorption has not been established [1]. Antioxidant compounds from L/F_300_ not absorbed through the intestinal wall would potentially be available to scavenge free radicals or be fermented by colonic bacteria and contribute to the overall antioxidant defence system of the GIT [1,51]. Further research is necessary to determine the fate of antioxidant compounds after absorption.

## 3. Experimental Section

### 3.1. Reagents

All chemicals used were “AnalaR” grade obtained from Sigma-Aldrich Ireland Ltd., Arklow, Co. Wicklow, Ireland and Merck KGaA, Darmstadt, Germany. Tissue culture plastics were supplied by Sarstedt, Wexford, Ireland and the Caco-2 cell line (Human Caucasian colon adenocarcinoma) were from the European Collection of Animal Cell Cultures, Wiltshire, UK. Fresh pork meat (*longissimus thoracis et lumborum* (LTL)) was supplied by Ballyburden Meat Processors, Ballincollig, Co. Cork, Ireland. Laminarin (L) (MW = 13 kDa) and fucoidan (F) (MW = 57 kDa) standards from Sigma-Aldrich were isolated from *Laminaria digitata* and *Fucus vesiculosus*, respectively. A spray-dried seaweed extract (L/F), containing laminarin and fucoidan was manufactured by Bioatlantis, Tralee, Co. Kerry, Ireland. The extract isolated from brown seaweed (*Laminaria digitata*) was prepared using an acid extraction technique, details of which are industrially-confidential. The extract contained 0.64% protein, 9.3% laminarin, 7.8% fucoidan, and 8.3% mannitol and further details are reported in Moroney *et al.* [15].

### 3.2. Measurement of the DPPH Free Radical Scavenging Activities of Seaweed Polysaccharides (L, F and L/F)

The 1,1-diphenyl-2-picrylhydrazyl (DPPH) free radical scavenging activity of L, F and L/F was measured using the method of Qwele *et al.* [52] with slight modifications. DPPH (0.2 mM, 3 mL) in methanol was added to 3 mL of L (1 and 10 mg/mL; L_1_ and L_10_), F (1 mg/mL; F_1_) and L/F (1 and 3 mg/mL; L/F_1_ and L/F_3_). Trolox C (1 mg/mL; Trolox), was used as a positive control. Tubes were mixed and incubated for up to 20 h at room temperature (~20 °C) in the dark. The assay control contained 3 mL distilled water and 3 mL of DPPH solution. Absorbance measurements were recorded spectrophotometrically (Cary 300 Bio, UV-Vis spectrophotometer, Varian Instruments, Palo Alto, CA, USA) against a distilled water blank after 1, 4 and 20 h at 517 nm. The DPPH free radical scavenging activity, expressed as a percentage of the assay control was calculated as follows:

% inhibition of DPPH = [1 − (absorbance of sample/absorbance of assay control)] × 100(1)

### 3.3. The Effect of Seaweed Polysaccharides on Lipid Oxidation in Pork Muscle Homogenates

Pork homogenates (25% w/v) were prepared by homogenising LTL (70 g) in buffer (210 mL) (0.12 M KCL 5 mM histidine, pH 5.5) on ice using an Ultra-turrax T25 homogeniser. L, F and L/F were solubilised in distilled water and added to LTL homogenates at final concentrations of 3 and 6 mg/mL (L_3_, L_6_, F_3_, F_6_, L/F_3_ and L/F_6_) homogenate. Lipid oxidation in muscle homogenate samples (20 g) held at 4 °C was initiated by the addition of 45 μM FeCl_3_/sodium ascorbate (1:1). Muscle homogenates with and without FeCl_3_/sodium ascorbate and without antioxidants (L, F and L/F) were run simultaneously as controls with each experiment. Lipid oxidation measurements were measured after 4 h in samples held at 4 °C.

#### Measurement of Lipid Oxidation in Pork Muscle Homogenates

A modification of the 2-thiobarbituric acid (TBA) assay of Siu & Draper [53] was used to measure lipid oxidation in pork muscle (LTL) homogenates. Homogenate samples (4 mL) were added to 4 mL 10% trichloroacetic acid (TCA) and centrifuged (Beckman J2-21, Beckman Instruments Inc., Brea, CA, USA) at 6160× *g* for 15 min at 4 °C. Following centrifugation, the supernatant was filtered through Whatman No. 1 filter paper. In a screw cap test tube, the clear filtrate (4 mL) was added to 0.06 M TBA reagent (1 mL) and incubated at 80 °C for 90 min. The absorbance of the resulting coloured complex was measured using a spectrophotometer (Cary 300 Bio) at 532 nm against a blank containing buffer (2 mL, 0.12 M KCL 5 mM histidine, pH 5.5), 10% TCA (2 mL) and 0.06 M TBA reagent (1 mL). Results were expressed directly as absorbance values at 532 nm.

### 3.4. The Effect of Seaweed Polysaccharides on Oxymyoglobin Oxidation

#### 3.4.1. Preparation of Commercial Oxymyoglobin

Commercial horse heart oxymyoglobin (OxyMb) was prepared according to a modification of the method of Brown & Mebine [54]. Metmyoglobin (MetMb) (0.06 g) was dissolved in ice-cold distilled water (2 mL) to a concentration of 30 mg/mL and reduced to OxyMb by the addition of sodium dithionite at 1 mg/mL. To remove excess dithionite, OxyMb solution (2 mL) was applied to a glass column (2 cm i.d. × 25 cm) containing 10 g of mixed bed ion exchange resin (Amberlite MB-1A) and eluted from the column with approximately 20 mL cold distilled water. The OxyMb solution was passed through the column three times to reduce the conductivity to that of distilled water and was adjusted to a final volume of 50 mL with double strength buffer (300 mM KH_2_PO_4_-KOH, pH 5.5). The concentration of OxyMb in the final solution was calculated from its absorbance value at 525 nm divided by a millimolar extinction coefficient of 7.6 mM^−1^·cm^−1^ [55].

#### 3.4.2. Effect of Seaweed Polysaccharides on Oxymyoglobin Oxidation

Incubates (7 mL) containing OxyMb (~1 mg/mL) and L, F and L/F at two levels (0.1 and 1 mg/mL; L_0.1_, L_1_, F_0.1_, F_1_, L/F_0.1_ and L/F_1_) in 150 mM KH_2_PO_4_-KOH, pH 5.5, were prepared. Distilled water was used to prepare seaweed polysaccharide solutions (20 mg/mL). Additions to each OxyMb incubate were at a final concentration of 5% (v/v). Incubates were held at 4 °C and OxyMb oxidation was measured on days 0, 4 and 8 of storage.

Following centrifugation at 6160× *g* for 10 min at 4 °C, the absorbance spectra of the incubates (2 mL) containing commercial OxyMb were measured on a spectrophotometer (Cary 300 Bio) and spectral scans were recorded from 750 to 500 nm. The relative proportion of OxyMb (% of total myoglobin) was calculated using absorbance measurements at selected wavelengths (572, 565, 545 and 525 nm) as described by Krzywicki [55].

### 3.5. Effect of Cooking on DPPH Free Radical Scavenging Activity of Seaweed Polysaccharides in Pork Meat

Fresh minced LTL was assigned to one of five treatments: untreated pork (Control), L (100 mg/g pork; L_100_), F (100 mg/g; F_100_), L/F (100 mg/g; L/F_100_) and L/F (300 mg/g; L/F_300_). The levels of L, F and L/F added to fresh minced LTL were based on the DPPH free radical scavenging activities of the seaweed polysaccharides determined in Section 2.2. L, F and L/F were dissolved in water, immediately added to fresh minced LTL (5% v/w) and mixed vigorously. Minced LTL (1 g portion) from each treatment was retained for measurement of DPPH free radical scavenging activity of fresh minced LTL prior to cooking. The remaining fresh LTL (5 g portions) of each treatment were placed on aluminium foil lined trays and cooked at 180 °C for 5 min 30 s in a fan-assisted convection oven (Zanussi Professional, Model 10 GN1/1, Conegliano, Italy) until an internal temperature of 72 °C was reached.

Fresh and cooked minced LTL (1 g) were homogenised in 0.05 M phosphate buffer (9 mL), pH 7, using an Ultra Turrax T25 homogeniser and homogenates were centrifuged (Beckman J2-21) at 7800× *g* for 10 min at 4 °C. The supernatant fraction obtained (fresh/cooked minced LTL) was used for the measurement of the DPPH free radical scavenging activity [52]. DPPH (0.2 mM, 3 mL) prepared in methanol was added to 0.3 mL supernatant and 2.7 mL distilled water. The mixture was vortexed and left to stand at room temperature (~20 °C) in the dark. The assay control contained 0.3 mL phosphate buffer and 2.7 mL distilled water and 3 mL of DPPH solution. The absorbance of the solution was measured against a distilled water blank after 1, 4 and 20 h at 517 nm. The scavenging activity of the pork meat against the DPPH radical before and after cooking was expressed as a percentage of the assay control and calculated as:

% inhibition of DPPH = [1 − (absorbance of sample/absorbance of assay control)] × 100(2)

### 3.6. Effect of in Vitro Digestion on the DPPH Free Radical Scavenging Activity of Seaweed Polysaccharides in Cooked Pork Meat

The *in vitro* digestion procedure was adapted from that previously described by Daly *et al.* [56]. All experimental work was carried out in UV-light free conditions to reduce the possible photo-decomposition of L, F and L/F present in the cooked minced LTL. Briefly, cooked minced LTL (1 g) from each treatment were weighed into 100 mL plastic tubes and homogenized using an Ultra Turrax T25 homogeniser at 24,000 rpm for 10 s in 8 mL Hanks Balance Salts Solution (HBSS) containing BHT. HBSS (5 mL) was slowly pipetted down the homogeniser to rinse remaining residue into the plastic tubes. The homogenates were transferred into amber bottles (rinsed twice using 5 mL HBSS). In order to mimic the gastric phase of digestion, pepsin (1 mL) (0.04 g/mL in 0.1 N HCl) and HBSS (2 mL) was added and the pH was adjusted to 2 using 1 M HCl. Oxygen was displaced by blowing nitrogen over the samples for 5 s. Samples were then incubated at 37 °C for 1 h in an orbital shaking (95 rpm) water bath (Grant OLS200, Keison Products; Essex, UK).

After gastric digestion, the pH was increased to 5.3 using sodium carbonate (0.9 M NaHCO_3_) followed by the addition of 200 μL bile salts (1.2 mg/mL glycodeoxycholate, 0.8 mg/mL taurocholate and 1.2 mg/mL taurodeoxycholate) and 100 μL pancreatin (0.08 g/mL HBSS). Subsequently, the pH was increased to 7.4 using NaOH, oxygen was displaced by nitrogen and samples were incubated at 37 °C in the orbital shaking water bath for a further 2 h. Following intestinal digestion, the digested minced LTL (digestates) from each treatment were centrifuged (Beckman J2-21) at 7800× *g* for 10 min at 4 °C. Undigested minced LTL samples were diluted using HBSS to the same final volume as the digestates and subsequently centrifuged at 7800× *g* for 10 min at 4 °C.

The supernatant (aqueous fractions) of the undigested minced LTL and digestate samples were frozen at −80 °C until required for measurement of DPPH free radical scavenging activity (described in Section 3.5). The assay control contained 0.3 mL HBSS buffer and 2.7 mL distilled water and 3 mL of DPPH solution. The absorbance of the solution was measured against a distilled water blank after 1, 4 and 20 h at 517 nm. The scavenging activity of the pork meat against DPPH radical post digestion was corrected for the meat control and expressed as:

% inhibition of DPPH = [(1 − (Ab_sample_/Ab_ac_)) × 100] − [(1 − (Ab_meatcontrol_/Ab_ac_)) × 100] (3)

where Ab_sample_ = absorbance of sample; Ab_ac_ = absorbance of assay control; Ab_meatcontrol_ = absorbance of meat control.

### 3.7. Bioaccessibility and Theoretical Cellular Uptake of the Aqueous Fraction of Digested Minced LTL

Caco-2 cells were maintained in Dulbecco’s modified Eagle’s medium (DMEM), containing 10% (v/v) foetal bovine serum (FBS) and 1% (v/v) non-essential amino acids. Cells were grown at 37 °C /5% CO_2_ in a humidified incubator and were cultured with 0.5% Penicillin-Streptomycin (5000 U/mL). Cultures of Caco-2 cells were used between passages 46–51. To establish the Caco-2 intestinal model, the cells were seeded at a density of 6 × 10^4^ cells cm^−2^ on a transwell plate (12-well plate, 22 mm diameter, 0.4 µm pore size membrane). Media was changed every 2–3 days and experiments were performed when monolayers were 17–20 days post-confluency. The aqueous fraction of the digestates (control and L/F_300_) (125 µL) were diluted to a final volume of 500 µL with serum free media and added to the top chamber of the transwell plate. Serum free media (1 mL) was added to the basolateral chamber and the cells were incubated for 4 and 20 h. Preliminary work showed that the aqueous fraction of the digestates was not toxic to the cells (data not shown). The transepithelial electrical resistance (Millicell-ERS, Millipore, Cork, Ireland) was measured before and after the addition of the aqueous fraction of the digestates to ensure the monolayer remained intact. The media from the basolateral chamber was then harvested for the measurement of the DPPH free radical scavenging activity (see Section 3.5).

The assay control contained 0.3 mL serum free media and 2.7 mL distilled water and 3 mL of DPPH solution. The absorbance of the solution was measured against a distilled water blank after 4 h at 517 nm. The difference between the DPPH free radical scavenging activities of L/F_300_ and the control, expressed as a percentage of the control, was calculated for the aqueous fraction of the digestate (AF) and the transwell basolateral chamber media (TW) as follows:

% theoretical cellular uptake of antioxidant compounds = [(AF_L/F300_ − AF_meatcontrol_)/AF_meatcontrol_) × 100] − [(TW_L/F300_ − TW_meatcontrol_)/TW_meatcontrol_) × 100] (4)

where AF_L/F300_ = absorbance of aqueous fraction of the digestate L/F_300_; AF_meatcontrol_ = absorbance of aqueous fraction of the digestate meat control; TW_L/F300_ = absorbance of transwell basolateral chamber media following incubation of L/F_300_ with Caco-2 cells; TW_meatcontrol_ = absorbance of transwell basolateral chamber media following incubation of the meat control with Caco-2 cells. The difference in activity between AF and TW was attributed to theoretical uptake of antioxidant compounds by the Caco-2 cells.

### 3.8. Statistical Analysis

Each experiment was carried out three individual times. All analyses were performed in duplicate. The DPPH free radical scavenging activities of L, F and L/F, fresh and cooked LTL pork muscle, cooked LTL digestates and lipid oxidation mean values were analysed by one-way ANOVA. Means were considered significantly different at (*p* < 0.05) using Tukey’s post hoc test. A full repeated measures ANOVA was conducted to investigate the effects of L, F and L/F concentration and time on oxymyoglobin oxidation. L, F and L/F represented the “between-subjects” factor and the effect of time was measured using the “within-subjects” factor. Tukey’s test was used to adjust for multiple comparisons between treatment means (*p* < 0.05). All analysis was carried out using the SPSS 18.0 for Windows (SPSS, Chicago, IL, USA) software package.

## 4. Conclusions

Due to the presence of sulphate groups and anionic charge, fucoidan is a more potent free radical scavenging antioxidant than laminarin. Furthermore fucoidan is at least, in part, responsible for the antioxidant activity observed by the L/F extract in previous studies. Fucoidan may be a potential natural antioxidant to enhance lipid stability in meat products. The antioxidant potential of fucoidan and the L/F extract is strongly influenced by the cooking and digestion processes. The L/F extract demonstrated superior antioxidant activity compared to fucoidan in minced LTL, after cooking and post digestion. The antioxidant compounds of the L/F extract were partially absorbed by Caco-2 cells confirming their bioaccessibility post digestion. Results demonstrate the potential for extracts containing fucoidan to enhance antioxidant activity of functional cooked meat products as well as contribute to human antioxidant defence systems.
